# Combined HER2‐targeted mRNA‐LNP vaccine and radiotherapy suppress Lewis lung carcinoma growth in mice: Efficacy and mechanistic insights

**DOI:** 10.1002/btm2.70160

**Published:** 2026-07-20

**Authors:** Shuo Wang, Shaowu Jing, Suyang Zou, Yao Zhang, Mingming Ge, Kangqi Gao, Xinyi Li, Lianmei Zhao, Jun Wang

**Affiliations:** ^1^ Department of Radiation Oncology The Fourth Hospital of Hebei Medical University, Hebei Clinical Research Center for Radiation Oncology Shijiazhuang China; ^2^ Department of General Surgery The Second Hospital of Hebei Medical University Shijiazhuang China; ^3^ Scientific Research Center The Fourth Hospital of Hebei Medical University Shijiazhuang China

**Keywords:** HER2, Lewis lung carcinoma, mRNA‐LNP vaccine, radiotherapy, tumor immune microenvironment

## Abstract

HER2 overexpression is a pivotal oncogenic alteration in NSCLC and is associated with poor prognosis. Existing HER2‐targeted therapeutics, including antibody–drug conjugates, have limited anticancer efficacy and prominent systemic toxicity, whereas radiotherapy‐mediated tumor immunosuppression further hinders therapeutic outcomes. This study aimed to construct a HER2‐targeted mRNA vaccine delivered by lipid nanoparticles (LNPs), designated HER2‐mRNA‐LNP, and to explore its standalone or combined anti‐tumor effects alone or in combination with radiotherapy (RT) in HER2‐overexpressing NSCLC xenografts, as well as the underlying immune regulatory mechanisms. We first prepared the HER2‐mRNA‐LNP vaccine and constructed HER2‐overexpressing Lewis lung carcinoma (LLC) cell line (HER2‐LLC) to establish matching tumor‐bearing xenograft mice. Tumor‐bearing mice were divided into four groups: LNP control, HER2‐mRNA‐LNP, RT, and HER2‐mRNA‐LNP + RT (combination). Prophylactic and therapeutic effects were assessed by measuring tumor volume and weight, performing CCK‐8 cytotoxicity assays, immunohistochemistry (HER2, Ki67), and flow cytometry for immune cell profiling. Our results demonstrated that HER2‐mRNA‐LNP significantly reduced tumor volume and weight compared with LNP controls and this suppression was further enhanced when combined with RT. Expression of HER2 and Ki67 decreased in both the HER2‐mRNA‐LNP and combination groups. HER2‐mRNA‐LNP increased the proportions of CD8^+^ T cells, NK cells, B cells, and M1 macrophages in tumors and spleens, elevated levels of IFN‐γ, TNF‐α, IL‐4, and IL‐2 in splenic lymphocytes, and reduced the proportion of regulatory T cells (Tregs) in tumor tissues. Combination with RT further augmented this immune remodeling. Collectively, prophylactic administration of HER2‐mRNA‐LNP provided safe and effective antigen‐specific protection in mice and sensitized tumors to radiotherapy. Therapeutic administration of HER2‐mRNA‐LNP combined with RT exerted an enhanced antitumor effect on HER2‐LLC xenografts. This effect may be mediated by the induction of a mixed Th1/Th2 immune response and by modulation of both the tumor immune microenvironment and systemic immunity.

**Translational Impact Statement**

This study demonstrates for the first time that the combination of HER2‐mRNA‐LNP vaccine and radiotherapy remodels the tumor immune microenvironment, offering a novel therapeutic strategy for HER2‐overexpressing NSCLC. These findings establish a foundation for advancing the clinical translation of mRNA vaccine‐based radio‐immunotherapy and may improve the prognosis of patients with refractory HER2‐positive lung cancer.

## 1 BACKGROUND

Human epidermal growth factor receptor 2 (HER2), a key member of the EGFR family, is a transmembrane glycoprotein with tyrosine kinase activity.^1^, ^2^ Its structure comprises an extracellular domain (ECD), a transmembrane domain (TM), and an intracellular tyrosine kinase domain (TKD).^3^ HER2 alterations—including mutations, amplification, and overexpression—are closely associated with malignant behaviors such as tumor proliferation, metastasis, and therapy resistance, establishing HER2 as a critical therapeutic target in NSCLC, breast cancer, and gastric cancer.^4^, ^5^, ^6^, ^7^, ^8^ Characterized by high aggressiveness and poor response to treatment, HER2‐positive lung cancer represents a major clinical dilemma. Approximately 7.7%–23% of NSCLC patients exhibit HER2 overexpression^9^, ^10^; these individuals often respond poorly to conventional chemotherapy and targeted therapies, resulting in unfavorable prognoses. Although antibody–drug conjugates (ADCs) like trastuzumab deruxtecan (T‐DXd) show promise in HER2‐positive lung cancer,^11^, ^12^ current NCCN guidelines recommend T‐DXd only for HER2‐mutant NSCLC. The DESTINY‐Lung01 study demonstrated T‐DXd's efficacy in HER2‐overexpressing NSCLC but reported a high incidence of grade ≥3 treatment‐related adverse events and treatment‐related deaths.^13^ Currently approved only for later‐line treatment, T‐DXd faces challenges regarding systemic toxicity and acquired resistance.^14^, ^15^, ^16^, ^17^ Thus, safe and effective treatment strategies for HER2‐overexpressing NSCLC are urgently needed.

NSCLC involves multi‐step genetic alterations, including driver gene mutations and inactivation of tumor suppressor genes. During disease progression, immune homeostasis shifts toward tumor immune evasion alongside the formation of an immunosuppressive tumor microenvironment (TME), which ultimately leads to malignant cell proliferation. Immunotherapy exerts antitumor effects by reversing tumor‐induced immune suppression and activating endogenous T cells to eliminate tumor cells. HER2 overexpression, which defines a critical molecular subtype of NSCLC, drives malignant progression through persistent activation of the PI3K/AKT and MAPK signaling pathways, and tumors with HER2 overexpression exhibit primary or acquired resistance to conventional anti‐HER2 therapies. Owing to its accessible extracellular domain (ECD), HER2 serves as a classic immunotherapy target and is well suited to mRNA vaccine development.^18^, ^19^

Early research and development efforts for HER2‐related vaccines have mainly focused on protein/peptide vaccines and DNA vaccines. However, clinical translation has been persistently hampered by insufficient immunogenicity, suboptimal antigen presentation efficiency, and host immune tolerance. The advent of mRNA vaccine technology has opened new avenues for precision tumor immunotherapy. Crosby et al.^20^ reported a phase I clinical trial of an alphavirus replicon particle‐based HER2 vaccine (VRP‐HER2) in patients with recurrent breast cancer, demonstrating favorable tolerability, with antitumor effects observed in 7 of 22 patients and long‐term survival in 2 cases, thereby providing preliminary evidence for the clinical feasibility of HER2‐targeted vaccination. Nevertheless, this study employed a viral vector platform, and issues pertaining to delivery stability and adverse effects remain unresolved. Recent innovations in lipid nanoparticle‐delivered mRNA vaccines (mRNA‐LNP vaccines) are expected to overcome these limitations.^21^, ^22^ By delivering mRNA encoding target antigens via LNPs, these vaccines efficiently activate antigen‐presenting cells (APCs) in vivo, inducing antigen‐specific T and B cell immune responses.^23^, ^24^ Compared to traditional vaccines, the mRNA‐LNP platform allows rapid development, flexible design, potent activation of Th1 and cytotoxic T lymphocyte (CTL) responses, and enhanced safety due to non‐integration into the host genome.^25^ Lipid nanoparticles (LNPs) encapsulate HER2‐encoding mRNA to protect it from nuclease degradation. The formulation efficiently delivers mRNA into target cells such as dendritic cells and somatic cells, where the full‐length HER2 ECD protein is translated to achieve in situ antigen expression. The translated HER2 antigen is processed into short peptides by the proteasome and presented via MHC class I and MHC class II molecules. This process activates HER2‐specific CD8^+^ cytotoxic T cells and triggers the production of HER2‐specific antibodies to exert antitumor effects. As a self‐antigen, HER2 tends to induce immune tolerance in the host. Through more efficient presentation, the mRNA‐LNP vaccine can effectively break such tolerance and elicit stronger and more sustained immune responses.

Therefore, we constructed a HER2‐targeted mRNA vaccine using a stable LNP platform for delivery, with the aim of investigating superior intervention strategies. Extensive preclinical studies and numerous Phase I/II clinical trials are underway for mRNA‐LNP vaccines in breast cancer, colorectal cancer, melanoma, and NSCLC.^26^

The mature development and delivery technology of mRNA‐LNP provide a solid foundation for this study. However, no vaccine specifically targeting HER2‐overexpressing NSCLC has yet entered clinical trials. This research gap underscores the urgency and innovation of developing HER2‐targeted mRNA‐LNP vaccines.

Radiotherapy, a cornerstone of local lung cancer treatment, exhibits immune‐modulatory functions beyond local tumor killing.^27^, ^28^, ^29^ However, its efficacy is often limited by the immunosuppressive tumor microenvironment. Vaccines can extend RT‐induced local immunity to a systemic level. By modulating the tumor immune microenvironment, the combination of vaccine and RT may cooperatively enhance antitumor immunity and alleviate immunosuppression.^30^ Notably, for both prophylactic and therapeutic vaccines, the antigen pool released by RT may act together with pre‐existing or vaccine‐induced antigens,^31^, ^32^ thereby amplifying the immune response. Concurrently, vaccine‐ and RT‐induced immune changes can mutually enhance their efficacy. This combined effect holds promise for overcoming the dual challenges of local control and systemic defense in lung cancer treatment. To our knowledge, nanoparticle‐based vaccine delivery combined with RT remains unexplored in HER2‐overexpressing NSCLC, representing a key innovation of this study. We investigated the response of HER2‐LLC xenografts to RT in vaccine‐protected mice and evaluated the therapeutic efficacy of the vaccine combined with RT in established HER2‐LLC tumors, thereby highlighting the innovative dual application of vaccine and RT at different treatment time points and integrating both preventive and therapeutic aspects of cancer vaccine research.

This study aimed to develop a HER2‐targeted mRNA‐LNP vaccine (HER2‐mRNA‐LNP). We evaluated its protective effect against HER2‐overexpressing Lewis lung carcinoma (HER2‐LLC) xenografts and its radiosensitizing effect when combined with RT. We also assessed the therapeutic efficacy of HER2‐mRNA‐LNP against established HER2‐LLC xenografts and its combined effect with RT, further investigating the associated immune mechanisms. Our goal is to provide a reference and rationale for future research and the design of safe, effective clinical strategies.

## 2 MATERIALS AND METHODS

### 2.1 Animals

Six 6‐week‐old adult male BALB/c mice and forty‐six 6‐week‐old adult male C57BL/6 mice were obtained from the Laboratory Animal Center of Hebei Medical University (License No. SCXK (Jing) 2024‐0003). All animal experiments were approved by the Ethics Committee of the Fourth Affiliated Hospital of Hebei Medical University (Approval No. IACUC‐4th Hos Hebmu‐No. 2022016‐2).

### 2.2 Construction of HER2‐LLC cell line

The mouse Lewis lung carcinoma cell line LL/2 (commonly abbreviated as LLC; RRID: CVCL_4358) was kindly provided by Hebei Medical University in 2024. The cell line was originally derived from lung tissue of a male C57BL/6 mouse. Prior to experimentation, its identity was authenticated as LLC through short tandem repeat (STR) profiling, and it was confirmed to be free of mycoplasma contamination using the MycoAlert™ PLUS Detection Kit (Lonza).

A full‐length HER2 plasmid was stably integrated into the mouse LLC genome using a transposon‐mediated gene delivery system. A stable HER2‐overexpressing cell line (HER2‐LLC) was selected. HER2 overexpression was confirmed at the RNA and protein levels by quantitative PCR (Q‐PCR) and Western blot, respectively.

### 2.3 Preparation of HER2‐mRNA‐LNP vaccine

Based on previous work developing rat and human HER2 ECD‐based vaccines, the human variant was selected as the model antigen due to its superior immunogenicity and enhanced antitumor efficacy against 4T1 breast cancer xenografts in mice. The sequence was verified by Sanger sequencing and agarose gel electrophoresis, and human HER2 ECD protein expression was confirmed by Western blot. Using plasmid DNA stably expressing human HER2 ECD as a template, mRNA was synthesized via in vitro transcription, capping, and polyadenylation. The classic MC3 formulation was used, with a molar ratio of DLin‐MC3‐DMA:DSPC:Cholesterol:DMG‐PEG_2000_ = 50:10:38.5:1.5 and an N/P ratio of 6:1. A microfluidic device was employed to thoroughly mix the organic ethanol phase with the aqueous mRNA phase.

### 2.4 Detection of mouse serum HER2 antibody levels by ELISA

Whole blood was collected, allowed to clot, and centrifuged to obtain serum. Human HER2 ECD (Sino Biological; 0.25 mg/mL) was coated onto an ELISA plate overnight at 20 ng/well. After washing with PBST, plates were blocked with 5% BSA at 37°C for 2 h. Diluted serum samples were added and incubated at 37°C for 1 h, followed by incubation with HRP‐conjugated goat anti‐mouse secondary antibody (Solarbio, 1:7000). After washing, color development was performed using TMB substrate for 5 min at 37°C in the dark. The reaction was stopped, and absorbance was measured at 450 nm.

### 2.5 Grouping and treatment

To evaluate the antibody titers induced by HER2‐mRNA‐LNP, BALB/c and C57BL/6 mice were assigned to the LNP and HER2‐mRNA‐LNP groups (*n* = 3 per group). After adaptive feeding, all mice were sequentially numbered and randomized using random numbers generated via Microsoft Excel. Investigators performing animal maintenance and baseline evaluations were blinded to group allocation to eliminate selection bias, and no significant differences in body weight or general health were detected among groups. Given that HER2‐LLC tumor formation was only achievable in C57BL/6 mice, this strain was utilized for all subsequent experiments. For prophylactic and therapeutic intervention assays, 20 C57BL/6 mice were randomly divided into four groups (*n* = 5 per group): the LNP, HER2‐mRNA‐LNP, RT, and HER2‐mRNA‐LNP + RT (combination) groups. Mouse randomization was conducted using a sequence generated by SPSS 26.0 software. No animal exclusion or dropout occurred throughout the experiment, and all groups exhibited consistent baseline characteristics. Operators and outcome assessors remained blinded to group assignments to reduce experimental bias. Mice in the prophylactic group received three injections of HER2‐mRNA‐LNP before tumor challenge, whereas those in the therapeutic group were treated after successful tumor establishment. Radiotherapy was performed after tumor formation. HER2‐mRNA‐LNP was delivered via intramuscular injection at a dose of 10 μg per mouse in a total volume of 100 μL per dose. The LNP control group received an equivalent volume of empty LNP. Injections were administered once every 2 weeks in all groups. X‐ray radiation (6 MV) was delivered via a Varian Clinac 23EX linear accelerator with standardized parameters: total radiation dose of 24 Gy delivered in three fractions of 8 Gy, irradiation field of 1.5 × 1.5 cm, penetration depth of 0.5 cm, dose rate of 4 Gy/min, gantry angle of 0°, source‐to‐skin distance of 100 cm, and lead block shielding. For tumor modeling, 1 × 10^6^ HER2‐LLC cells were subcutaneously inoculated into the right lower limb of each mouse.

### 2.6 Flow cytometry for immune cell proportions in spleen and tumor tissues

Single‐cell suspensions from spleens and tumors were prepared using a gentleMACS Dissociator (Miltenyi Biotec). After Fc receptor blocking, cells were stained with live/dead dye first, followed by surface staining for 30 min at 4°C in the dark. For intracellular staining, cells were permeabilized using BD permeabilization buffer prior to antibody incubation. All samples were filtered before analysis by flow cytometry.

### 2.7 ELISA for cytokine levels in splenic lymphocytes

Splenic lymphocytes were isolated and seeded at 1 × 10^5^ cells/well. Cells were stimulated with 1 μg/well human HER2 ECD for 24–72 h. Levels of IFN‐γ, TNF‐α, IL‐2, and IL‐4 in the supernatant were measured using ELISA kits (MULTI SCIENCES). Absorbance was read at 450 nm with a reference wavelength of 630 nm. Final OD values were calculated by subtracting the reference OD from the 450 nm measurement.

### 2.8 CCK‐8 cytotoxicity assay

HER2‐LLC cells were co‐cultured with splenic lymphocytes. CCK‐8 reagent was added to the cell suspension at a 9:1 ratio. OD values were measured at 0, 24, 48, and 72 h at 450 nm using a microplate reader.

### 2.9 H&E staining

At the study endpoint (day 70), mice were euthanized, and heart, liver, spleen, lung, and kidney tissues were immediately harvested, fixed in 4% paraformaldehyde for 24–48 h, embedded in paraffin, and sectioned at 5 μm. Tissue sections were dewaxed, hydrated, and stained with hematoxylin for 8 min, differentiated, and counterstained with eosin for 2 min. Sections were then dehydrated, cleared, mounted, and examined microscopically.

### 2.10 Immunohistochemistry

Tumor sections were deparaffinized, subjected to antigen retrieval, and blocked. Sections were incubated with anti‐HER2 (1:1000) and anti‐Ki67 (1:1000) primary antibodies at 4°C overnight. After incubation with HRP‐conjugated secondary antibody, DAB staining was performed. Integrated optical density (IOD) analysis was used to quantify HER2 and Ki67 expression.

### 2.11 Statistical analysis

An a priori sample‐size and statistical power analysis was performed using G*Power 3.1 prior to all animal experiments to ensure the reliability and validity of the in vivo results. The type I error rate (statistical significance level, *α*) was set at 0.05, and the statistical power (1 − *β*) was set at 0.8, which are the universally recognized standard thresholds in preclinical biomedical research. Computational results verified that the adopted sample size was adequate to detect statistically significant intergroup differences and effectively minimize the risk of false‐negative outcomes. All datasets were first examined for normal distribution and homogeneity of variance to satisfy the fundamental prerequisites for parametric statistical tests. All statistical approaches were strictly selected according to the experimental design, data characteristics, and research purposes.

Statistical comparisons between two independent groups were conducted using the unpaired two‐sample Student's *t*‐test. For analyses of single phenotypic endpoints across three or more independent groups, parametric or non‐parametric approaches were selected based on data distribution characteristics: one‐way analysis of variance (ANOVA) with Tukey's honestly significant difference (HSD) post‐hoc test was employed for normally distributed data, while the Kruskal–Wallis test with Dunn's post‐hoc test was used for non‐normally distributed data. Two‐way ANOVA was performed to evaluate datasets influenced by two independent variables, with appropriate post‐hoc testing conducted when significant main effects or interactions were detected.

In this study, two‐way ANOVA was mainly applied to analyze dynamic antibody titer data and longitudinal tumor growth data, which involve two factors: experimental group and time. Two‐way ANOVA can effectively evaluate the main effects of each factor and detect their potential group‐by‐time interaction effect, which is critical for identifying time‐dependent changes in therapeutic efficacy. When a significant interaction was observed, Tukey's multiple comparisons test was further conducted to clarify the exact differences between groups at specific time points. Tukey's test was selected due to its excellent performance in controlling the family‐wise error rate and its high suitability for studies with equal sample sizes in each group (*n* = 5), ensuring rigorous and reliable multiple comparison results. All two‐way ANOVA analyses in this study were two‐way repeated‐measures ANOVA.

Pearson correlation analysis was utilized to evaluate the linear correlation between two continuous quantitative variables.

Differences with *p* < 0.05 were considered statistically significant. The significance levels were defined as follows: *p < 0.05, **p < 0.01, ***p < 0.001, and ****p < 0.0001.

## 3 RESULTS

### 3.1 Results 1: Preparation and characterization of HER2‐mRNA‐LNP

The HER2 ECD plasmid was constructed and verified by restriction digestion (HindIII/EcoRI), agarose gel electrophoresis, and Sanger sequencing (Figure 1a–c). Successful linearization was confirmed by a single mRNA band (Figure 1d). Blank LNPs displayed an average particle size of 60 ± 5.3 nm with a narrow size distribution (span = 0.22). After mRNA loading, the resulting HER2‐mRNA‐LNP exhibited a median diameter (D50) of 71 nm with D10 of 58 nm and D90 of 108 nm, corresponding to a span of 0.70, The polydispersity index (PDI) was 0.072, the *Z*‐average particle size was 79 ± 25.5 nm, and the zeta potential of 11.05 ± 2.97 mV (Figure 1e). Cryo‐transmission electron microscopy analysis revealed that the prepared HER2‐mRNA‐LNPs exhibited uniform, regularly spherical morphology, with no obvious particle aggregation or structural damage. Notably, in contrast to the few blank LNPs observed in the images, the mRNA‐loaded particles were uniformly distributed and contained dense mRNA cores (Figure 1f). The encapsulation efficiency was 80.1 ± 1.3% (Figure 1g). The relatively narrow span indicated good size uniformity of HER2‐mRNA‐LNP, which was consistent with a low polydispersity index typically observed for well‐formulated LNPs, suggesting high stability and suitability for in vivo delivery. Western blot confirmed HER2 ECD protein expression (Figure 1h). Immunization of male BALB/c and C57BL/6 mice with HER2‐mRNA‐LNP elicited high levels of specific antibodies, confirming its immunogenicity independent of LNP effects (Figure 1i).

**FIGURE 1:**
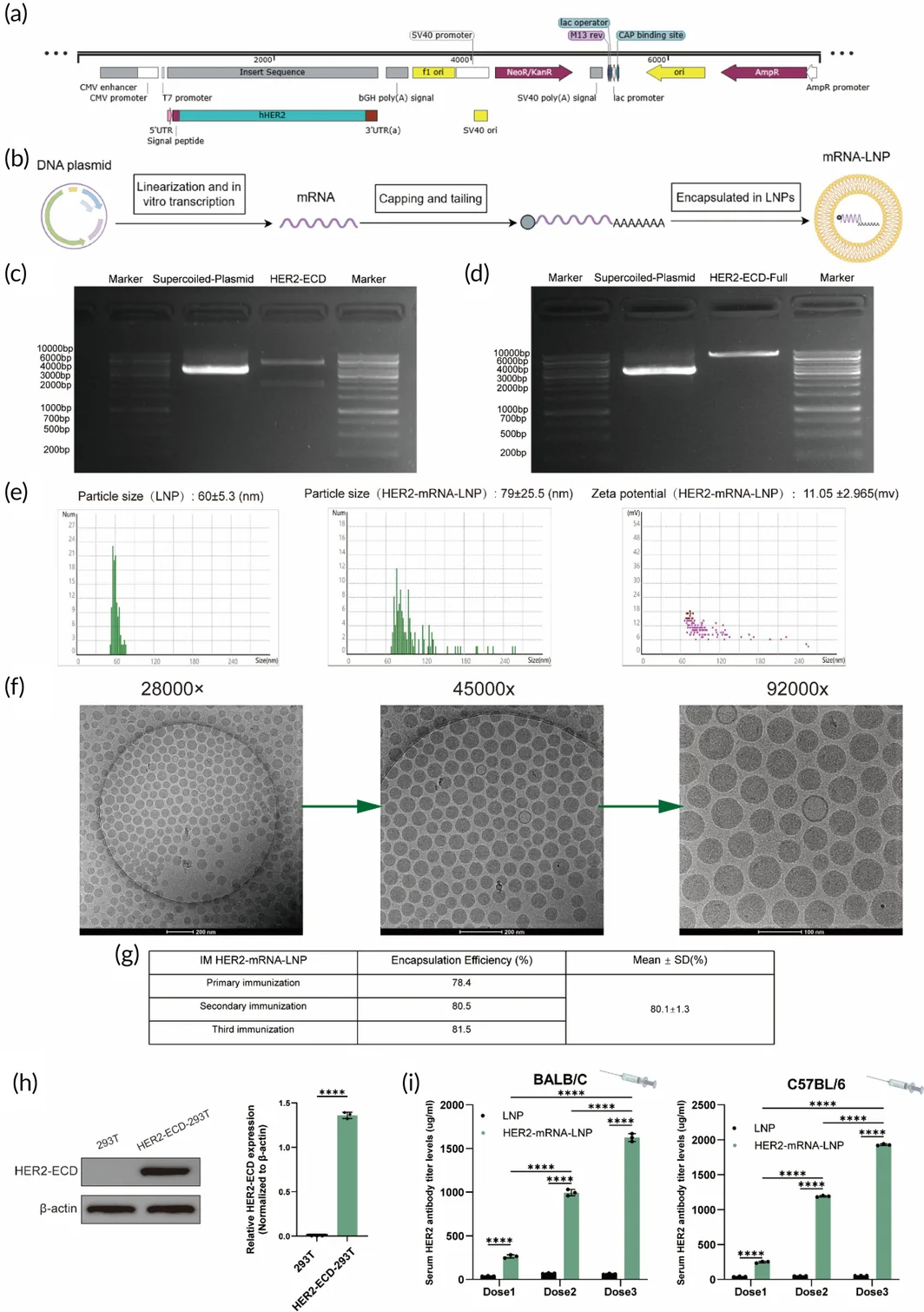
(a) Plasmid map of the HER2 extracellular domain (ECD). (b) Flowchart of HER2‐mRNA‐LNP preparation. (c) Agarose gel electrophoresis of HER2 ECD mRNA (double‐site digested product). (d) Agarose gel electrophoresis of HER2 ECD total mRNA (single‐site digested product). (e) Particle size and zeta potential of blank LNPs and HER2‐mRNA‐LNPs. (f) Representative cryo‐TEM micrographs of HER2‐mRNA‐LNPs at magnifications of 28,000×, 45,000×, and 92,000×. (g) Encapsulation efficiency of HER2‐mRNA‐LNPs. (h) Western blot analysis of HER2 ECD protein expression, with relative grayscale values quantified and statistically analyzed by Student's *t*‐test (*n* = 3 independent experiments). (i) Serum anti‐HER2 antibody titers in BALB/c and C57BL/6 mice immunized with LNP or HER2‐mRNA‐LNP after three doses. Data are presented as mean ± SEM. *n* = 3 mice per group. Statistical significance was determined by two‐way repeated‐measures ANOVA followed by Tukey's multiple comparisons test. ****p < 0.0001.

### 3.2 Results 2: Safety profile of HER2‐mRNA‐LNP

Following confirmation of immunogenicity, we evaluated the in vivo safety of HER2‐mRNA‐LNP. No significant macroscopic changes were observed in the livers, lungs, or hearts of HER2‐mRNA‐LNP‐treated mice compared with the LNP group, although spleen size was reduced (Figure 2a). Organ weights and body weight showed no notable differences except for decreased spleen weight in the HER2‐mRNA‐LNP group (Figure 2b), likely reflecting tumor‐induced compensatory adaptation rather than toxicity. H&E staining revealed no pathological alterations in the liver, lung, heart, or spleen (Figure 2c). Complete blood counts (WBC, RBC, PLT) and serum biochemical parameters—including liver function markers (ALT, AST, ALP, γ‐GT, ALB, DBIL, TBIL, and TBA), renal function markers (UREA, CREA, UA), and cardiac enzymes (CK, CK‐MB, LDH, and LDH1)—remained within normal ranges and were comparable to the LNP group and the PBS group (Figure 2d–g). These results demonstrate that HER2‐mRNA‐LNP exhibits a favorable safety profile without obvious adverse effects.

**FIGURE 2:**
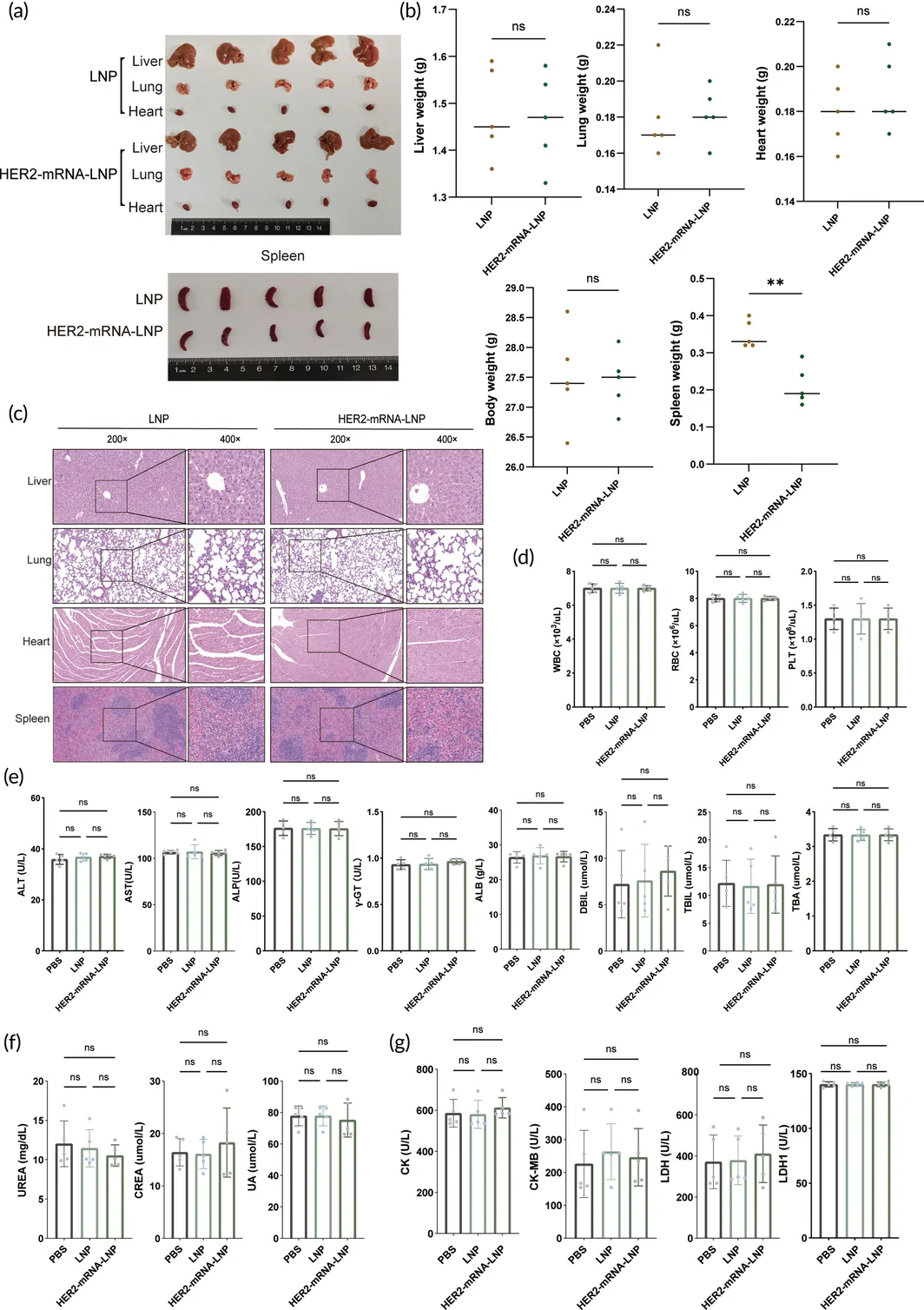
(a) Gross images of liver, lung, heart, and spleen from mice treated with LNP or HER2‐mRNA‐LNP. (b) Body weight and organ weights (liver, lung, heart, and spleen) of mice in the two groups. (c) H&E staining of liver, lung, heart and spleen tissue sections (magnification: 200× and 400×). (d) Peripheral blood routine analysis: white blood cell (WBC), red blood cell (RBC), and platelet (PLT) counts. (e) Serum liver function parameters: alanine transaminase (ALT), aspartate transaminase (AST), alkaline phosphatase (ALP), γ‐glutamyl transpeptidase (γ‐GT), albumin (ALB), direct bilirubin (DBIL), total bilirubin (TBIL), and total bile acid (TBA). (f) Serum renal function parameters: Urea (UREA), creatinine (CREA), and uric acid (UA). (g) Serum myocardial enzyme profile: creatine kinase (CK (U/L)), creatine kinase‐MB (CK‐MB), lactate dehydrogenase (LDH), and lactate dehydrogenase isoenzyme 1 (LDH1). All data are presented as mean ± SEM, with *n* = 5 mice per group. Statistical analysis was performed using unpaired two‐tailed Student's *t*‐test. **p < 0.01; ns, not significant (*p* > 0.05).

### 3.3 Results 3: Prophylactic effect of HER2‐mRNA‐LNP on HER2‐LLC xenografts and radiosensitization

To establish a clinically relevant HER2‐expressing murine tumor model, we first characterized HER2 mRNA and protein expression in multiple cell lines via Q‐PCR (*p* < 0.0001) and Western blot. Both assays confirmed significantly higher HER2 expression in HER2‐LLC than in parental LLC cells (*p* < 0.0001), verifying successful stable transfection. Moreover, compared with LLC cells, HER2‐LLC cells showed a markedly increased HER2 protein expression level (*p* < 0.0001). The expression level was intermediate between that of the human HER2‐positive breast cancer cell line SKBR3 and the human lung cancer cell line A549, which is consistent with the HER2 expression profile observed in clinical patients with NSCLC (Figure 3a). Antibody titers were substantially elevated in the HER2‐mRNA‐LNP and combination groups following three vaccine doses, whereas the LNP and RT alone groups generated negligible responses (all *p* < 0.0001; Figure 3b). Tumors formed in all groups within 7–10 days post‐inoculation. Tumor growth was slower in the HER2‐mRNA‐LNP and RT groups than in LNP controls and was further inhibited in the combination group. Significant effects were detected for the group × time interaction (*p* < 0.0001), the main effects of time and group, and the subject effect (*p* < 0.0001) (Figure 3c, Table 1). The results of Tukey's test are shown in Table S1, and demonstrate that tumor growth was significantly suppressed in both the HER2‐mRNA‐LNP and combination groups compared with the LNP control from Day 4 onward (*p* < 0.05–0.0001), with the combination group consistently showing the lowest tumor volumes throughout the 24‐day observation period. Notably, the combination group achieved significantly smaller tumor volumes than the RT monotherapy group from Day 5 onward (*p* < 0.05), whereas the HER2‐mRNA‐LNP and combination groups maintained comparable efficacy at all time points (all *p* > 0.05), indicating that the mRNA vaccine, rather than radiotherapy alone, was the primary driver of antitumor activity. The treatment timeline is outlined in Figure 3d. Tumor volumes in the HER2‐mRNA‐LNP, RT, and combination groups were significantly smaller than those in the LNP group (*p* < 0.0001). Similarly, tumor weights in the HER2‐mRNA‐LNP (*p* < 0.001) and RT (*p* < 0.01) groups were lower than in the LNP group, with the combination group showing the greatest reduction (*p* < 0.0001) (Figure 3e,f). In vitro, after 48 h of co‐culture of splenic T lymphocytes from HER2‐LLC xenograft mice with HER2‐LLC cells, cytotoxicity was significantly higher in the HER2‐mRNA‐LNP and RT monotherapy groups than in the LNP group (*p* < 0.01), with the combination group showing the highest cytotoxicity (*p* < 0.0001) (Figure 3g). This result was consistent with the in vivo trend. At 24, and 72 h, HER2‐mRNA‐LNP or RT alone also showed varying degrees of tumor cell inhibition. Notably, 40 days after the third immunization, antibody levels in the HER2‐mRNA‐LNP group remained high, indicating durable protection. A significant inverse correlation was observed between serum HER2 antibody titer and endpoint tumor volume (Pearson *r* = −0.8889, *p* = 0.0437; Figure 3h), with higher antibody levels associated with smaller tumors. Immunohistochemistry revealed the highest HER2 and Ki67 expression in the LNP group, indicating active proliferation. Expression was significantly lower in the HER2‐mRNA‐LNP and RT groups (*p* < 0.01), suggesting that both treatments alone inhibit tumor proliferation. The lowest expression in the combination group indicates that HER2‐mRNA‐LNP sensitizes tumors to RT (*p* < 0.0001) (Figure 3i,j). Meanwhile, IHC confirmed HER2 expression in vivo. Finally, we further compared the dynamic changes in antibody levels following one immunization versus three consecutive immunizations. A significant time × group interaction was observed, along with significant main effects of time and group, and a significant subject effect (all *p* < 0.0001). The results demonstrated that booster immunization could markedly increase antibody titers (Figure 3k, Table 2). Post‐hoc Tukey's comparisons (Table S2) revealed that both 1‐dose and 3‐dose HER2‐mRNA‐LNP vaccination significantly induced serum HER2 antibody production compared with PBS control from Day 15 onward (all *p* < 0.0001), with the 3‐dose group achieving progressively higher titers than the 1‐dose group from Day 30 onward (*p* < 0.0001). Detailed pairwise comparisons and descriptive statistics are provided in Table S2.

**FIGURE 3:**
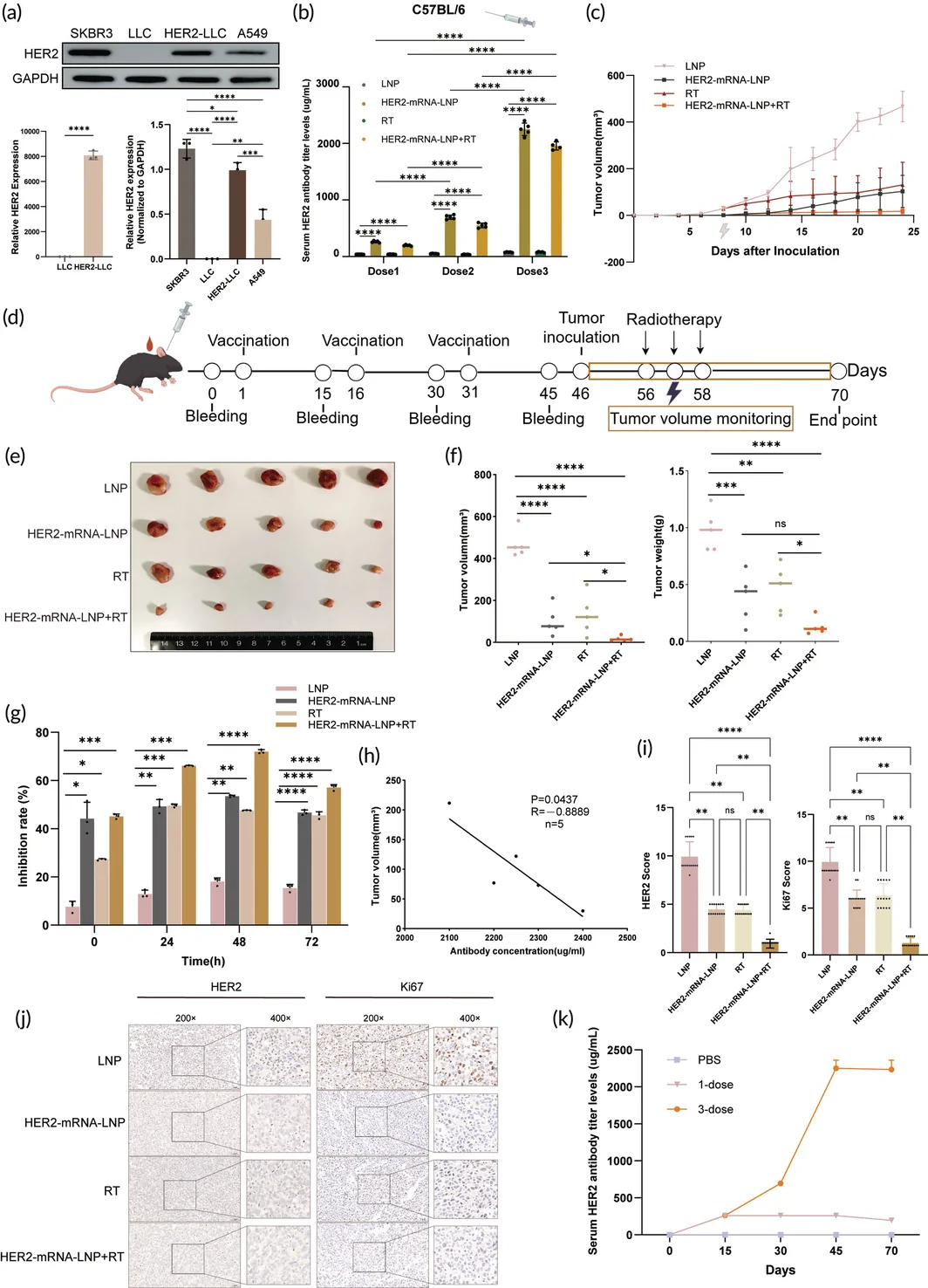
(a) HER2 mRNA expression in LLC and HER2‐LLC cells detected by Q‐PCR; HER2 protein expression in LLC, HER2‐LLC, SKBR3, and A549 cells analyzed by Western blot. (b) Anti‐HER2 antibody titers in mice after three doses of HER2‐mRNA‐LNP. (c) Tumor growth curve of HER2‐LLC tumor‐bearing mice in each group. (d) Treatment schedule for each group of mice. (e) Representative tumor images from each group. (f) Tumor volume and weight in each group. (g) CCK‐8 assay‐based inhibition rates of HER2‐LLC cells by splenic lymphocytes from tumor‐bearing mice. (h) Correlation analysis of tumor volume and antibody titer in HER2‐mRNA‐LNP group mice. (i) Quantitative immunohistochemical analysis of HER2 and Ki67 expression in tumor tissues from each group of mice. Three fields of view were selected per mouse, yielding 15 data points per group. (j) Representative immunohistochemical images of HER2 and Ki67 expression in mouse tumor tissues (magnification: 200× and 400×). (k) Kinetics of antibody titers over time in the PBS group, 1‐dose vaccine group, and 3‐dose vaccine group. All data are presented as mean ± SEM, with *n* = 5 mice per group. One‐way ANOVA with Tukey's test or Kruskal–Wallis test with Dunn's test was used for multiple group comparisons, as appropriate; statistical analysis was performed using two‐way repeated‐measures ANOVA followed by Tukey's multiple comparisons test for antibody titers, tumor growth curves and CCK‐8 inhibition rates; Pearson correlation analysis for correlation analyses (*R* = −0.8889, *p* = 0.0437). *p < 0.05, **p < 0.01, ***p < 0.001, ****p < 0.0001; ns, not significant (*p* > 0.05).

**TABLE 1:** Results of two‐way repeated‐measures ANOVA for tumor volume: preventive treatment.

Source of variation	Sum of squares	df	Mean square	*F*‐Value	*p*‐Value	Geisser–Greenhouse *ε*
Group × Time	1,034,895	36, 192	28,747	35.26	<0.0001	–
Time	1,057,388	1.458, 23.33	88,116	108.1	<0.0001	0.1215
Group	1,003,026	3, 16	334,342	34.98	<0.0001	–
Subject	152,926	16, 192	9558	11.72	<0.0001	–

*Note*: Two‐way repeated measures ANOVA was performed to assess the effects of groups (four groups) (*n* = 5), time, and their interaction on tumor volume, with subjects as the repeated measure. The Geisser–Greenhouse correction was applied for sphericity violation. –, not applicable. ****p < 0.0001.

**TABLE 2:** Results of two‐way repeated‐measures ANOVA for serum HER2 antibody titer levels.

Source of variation	Sum of squares	df	Mean square	*F*‐Value	*p*‐Value	Geisser–Greenhouse *ε*
Group × Time	15,092,925	8, 48	1,886,616	1663	<0.0001	–
Time	8,588,347	1.112, 13.34	2,147,087	1893	<0.0001	0.2780
Group	16,824,175	2, 12	8,412,088	1447	<0.0001	–
Subject	69,780	12, 48	5815	5.127	<0.0001	–

*Note*: Two‐way repeated measures ANOVA was performed with Geisser–Greenhouse correction for sphericity violation (*n* = 5). The analysis evaluated the effects of time (five time points), treatment group (three groups), and their interaction on serum HER2 antibody titer levels, with subjects as the repeated measure. –, not applicable. ****p < 0.0001.

### 3.4 Results 4: HER2‐mRNA‐LNP activates the tumor immune microenvironment and enhances the efficacy of RT

To investigate the effects of HER2‐mRNA‐LNP combined with radiotherapy (RT) on the immune microenvironment of HER2‐LLC xenografts, we performed flow cytometry analysis on tumor tissues.

Compared with the empty LNP group, the proportion of CD8^+^ T cells were significantly increased in both the HER2‐mRNA‐LNP group (*p* < 0.0001) and the RT group (*p* < 0.0001). The combination therapy group showed a further increase in CD8^+^ T cells compared with both monotherapy groups (*p* < 0.0001), and the CD8^+^/CD4^+^ T cell ratio in the combination group was significantly higher than that in both the empty LNP group and the monotherapy groups (*p* < 0.0001) (Figure 4a).

**FIGURE 4:**
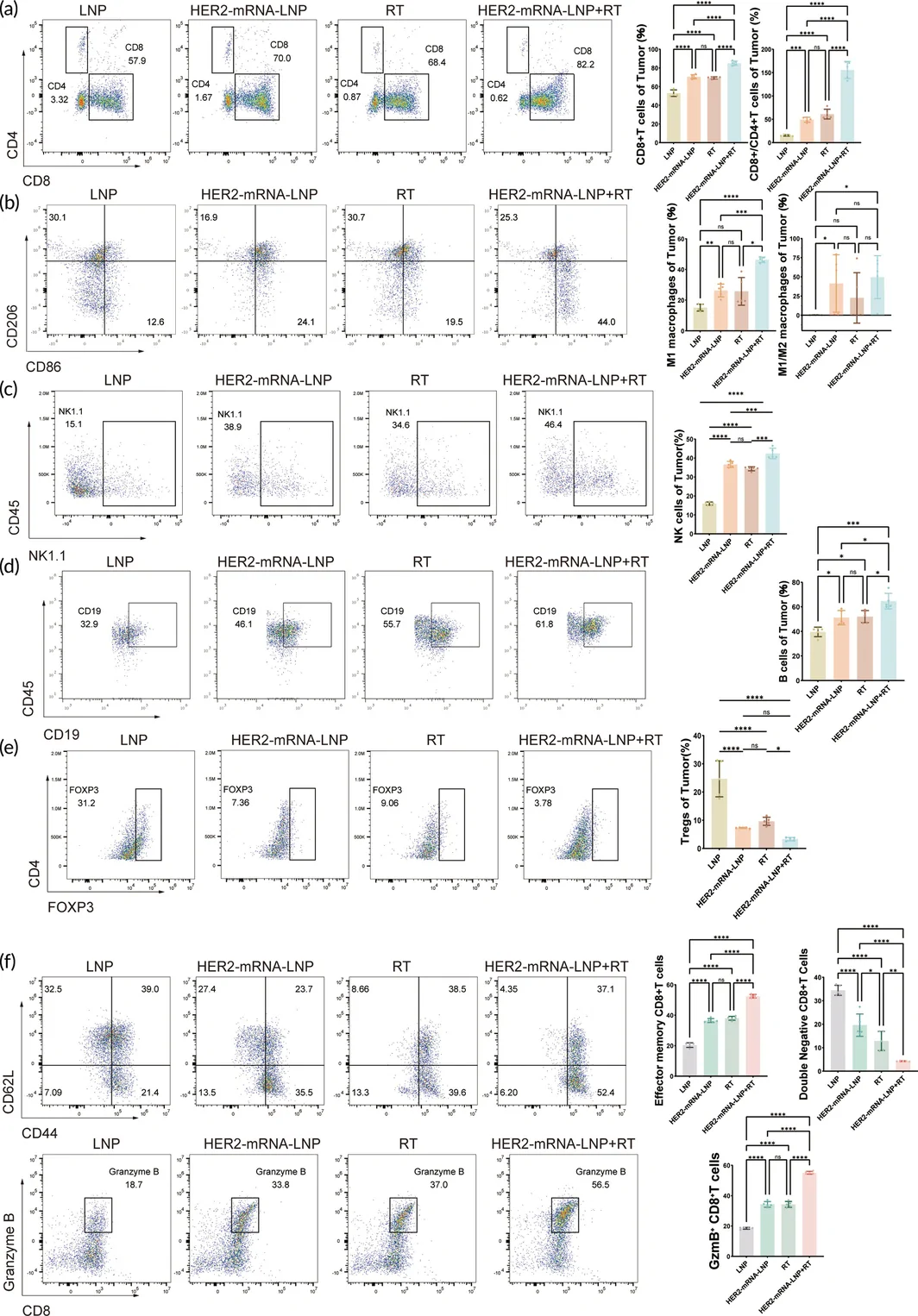
(a) Representative flow cytometry plots and quantification of CD8^+^ T cells and the CD8^+^/CD4^+^ T cells in tumor tissues from each group. (b) Representative flow cytometry plots and quantification of M1 macrophages and the M1/M2 ratio in tumor tissues from each group. (c) Representative flow cytometry plots and quantification of NK cell proportions in tumor tissues from each group. (d) Representative flow cytometry plots and quantification of B‐cell proportions in tumor tissues from each group. (e) Representative flow cytometry plots and quantification of Treg cell proportions in tumor tissues from each group. (f) Representative flow cytometry plots and quantification of effector memory T cells, double‐negative T cells, and Granzyme B^+^ T cells among CD8^+^ T cells in tumor tissues from each group. All data are presented as mean ± SEM, with *n* = 5 mice per group. One‐way ANOVA with Tukey's test or Kruskal–Wallis test with Dunn's test was used for multiple group comparisons. *p < 0.05, **p < 0.01, ***p < 0.001, ****p < 0.0001; ns, not significant (*p* > 0.05).

Analysis of macrophage polarization showed that, compared with the empty LNP group, the proportion of M1‐type macrophages increased by 1.5‐fold in the HER2‐mRNA‐LNP group and approximately 2.5‐fold in the combination group. The M1/M2 ratio of macrophages was 20‐fold higher in the HER2‐mRNA‐LNP group and 30‐fold higher in the combination group than in the empty LNP group (Figure 4b). These results indicate that HER2‐mRNA‐LNP promotes macrophage polarization toward the M1 phenotype, reversing the immunosuppressive tumor microenvironment, and that RT amplifies this effect.

Compared with the empty LNP group, the proportion of natural killer (NK) cells was significantly increased in the HER2‐mRNA‐LNP group (*p* < 0.0001), the RT group (*p* < 0.0001), and the combination group (*p* < 0.0001), with the combination group showing a further increase compared with the monotherapy groups (*p* < 0.001). The proportion of B cells was elevated in both the HER2‐mRNA‐LNP group and the RT group (*p* < 0.05), and further increased in the combination group (*p* < 0.001) (Figure 4c,d).

The proportion of regulatory T cells (Tregs) in all three treatment groups was significantly lower than that in the empty LNP group (*p* < 0.05), with a more significant reduction in the combination group compared with the RT group (*p* < 0.05) (Figure 4e).

To further characterize the activation status and functional phenotype of CD8^+^ T cells, we analyzed CD8^+^ T cell subsets. The proportion of CD8^+^ effector memory T cells (Tem, CD44^+^CD62L^−^) was significantly higher in all treatment groups than in the empty LNP group (*p* < 0.0001), and was further elevated in the combination group compared with the monotherapy groups (*p* < 0.0001) (Figure 4f). Consistently, the proportion of Granzyme B^+^ CD8^+^ T cells showed the same gradient increase across groups, with the highest percentage observed in the combination therapy group (*p* < 0.0001), indicating the strongest cytotoxic function of CD8^+^ T cells in this group. Meanwhile, the proportion of CD44^−^CD62L^−^ double‐negative (DN) T cells was significantly decreased in all treatment groups (*p* < 0.0001). The RT group showed a more significant reduction than the HER2‐mRNA‐LNP group (*p* < 0.05), and the combination therapy group exhibited a further decrease compared with both the HER2‐mRNA‐LNP group (*p* < 0.0001) and the RT group (*p* < 0.01). These findings reflect that the radiotherapy‐mediated bystander effect drives CD8^+^ T cells to differentiate into long‐lived effector memory phenotypes rather than transiently activated terminal effector cells. Collectively, these results demonstrate that HER2‐mRNA‐LNP combined with radiotherapy cooperatively induces both phenotypic and functional activation of CD8^+^ T cells, effectively enhancing cellular immune responses in the tumor microenvironment. The detailed gating strategy is shown in Figure S1.

### 3.5 Results 5: HER2‐mRNA‐LNP activates systemic immune responses and enhances the efficacy of RT

Flow cytometry analysis of splenic immune cells revealed increased proportions of CD8^+^ T cells and M1 macrophages in the HER2‐mRNA‐LNP (*p* < 0.05/*p* < 0.001) and RT (*p* < 0.05/*p* < 0.01) groups compared with the LNP group, with a more pronounced increase in the combination group (*p* < 0.0001). The CD8^+^/CD4^+^ T cell ratio (*p* < 0.001) and the M1/M2 ratio (*p* < 0.05) were both elevated in the combination group compared with the LNP group (Figure 5a,b). The proportion of NK cells was significantly increased in the HER2‐mRNA‐LNP (*p* < 0.0001) and RT (*p* < 0.0001) groups relative to the LNP group, with a further increase in the combination group (*p* < 0.0001). The proportion of B cells was elevated in all three treatment groups compared with the LNP group (*p* < 0.0001) (Figure 5c,d). ELISA analysis of cytokine levels in splenic lymphocyte supernatants revealed substantial increases in IFN‐γ (*p* < 0.05), TNF‐α (*p* < 0.001), IL‐2 (*p* < 0.01), and IL‐4 (*p* < 0.001) following vaccination, with further enhancement after combination with RT (*p* < 0.01–0.0001) (Figure 5e). The detailed gating strategy is shown in Figure S2.

**FIGURE 5:**
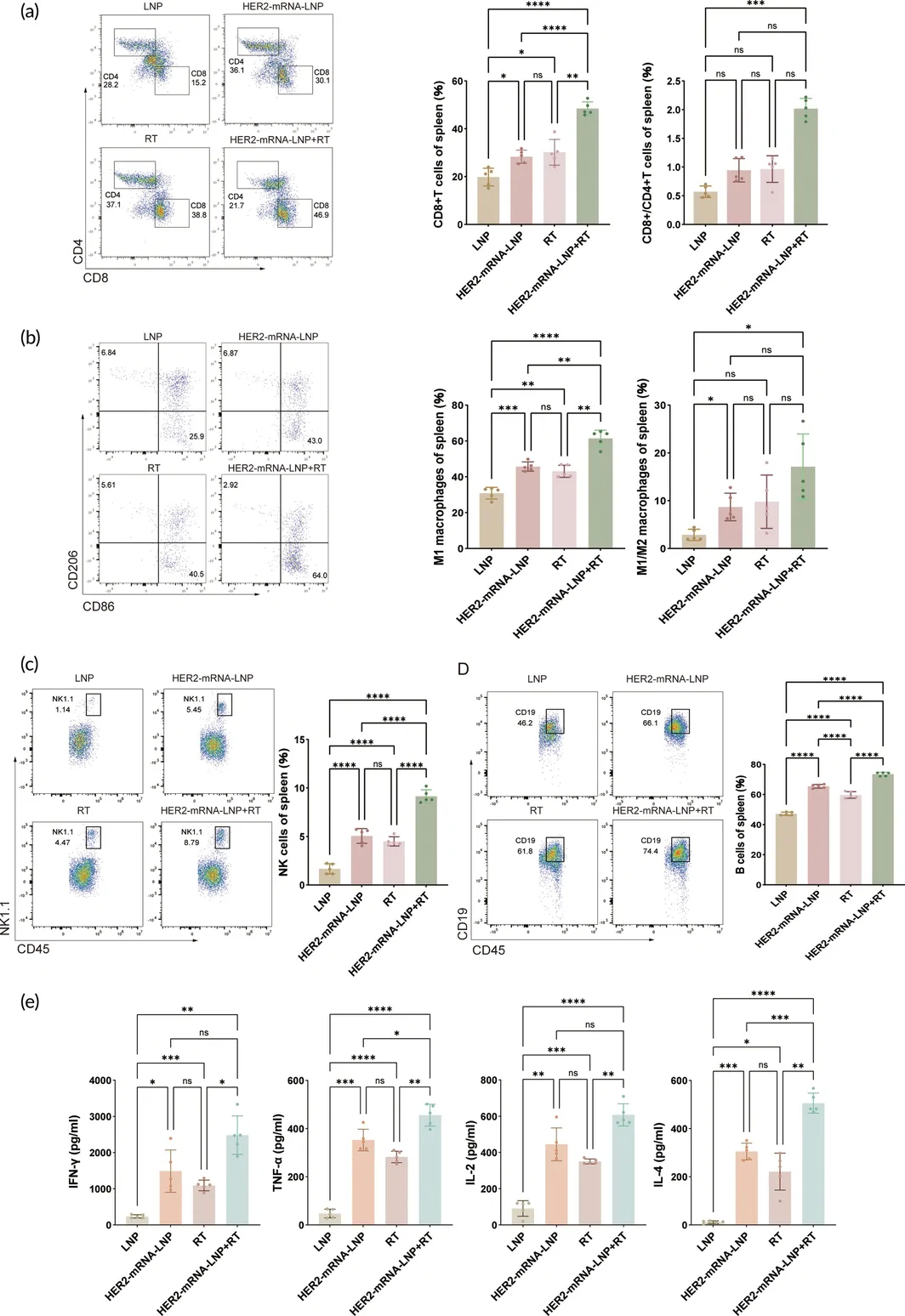
(a) Representative flow cytometry plots and quantification of CD8^+^ T cells and the CD8^+^ T/CD4^+^ T cell ratio in the spleens from each group. (b) Representative flow cytometry plots and quantification of M1 macrophages and the M1/M2 ratio in spleens from each group. (c) Representative flow cytometry plots and quantification of NK cell proportions statistics in spleens from each group. (d) Representative flow cytometry plots and quantification of B‐cell proportions in spleens from each group of mice. (e) Cytokine levels of IFN‐γ, TNF‐α, IL‐2, and IL‐4 in splenic lymphocyte supernatants were quantified. All data are presented as mean ± SEM, with *n* = 5 mice per group. One‐way ANOVA with Tukey's test or Kruskal–Wallis test with Dunn's test was used for multiple group comparisons. *p < 0.05, **p < 0.01, ***p < 0.001, ****p < 0.0001; ns, not significant (*p* > 0.05).

### 3.6 Results 6: Therapeutic efficacy of HER2‐mRNA‐LNP combined with RT

Finally, we evaluated the therapeutic efficacy of HER2‐mRNA‐LNP combined with RT. Tumors formed 7–10 days after HER2‐LLC cell inoculation. On day 14, mice received RT followed by vaccination (experimental timeline: Figure 6a). Tumor growth was slower in the HER2‐mRNA‐LNP and RT groups compared with the LNP group, with the most significant inhibition observed in the combination group. Significant effects were detected for the time × group interaction, the main effects of time and group, and the subject effect (all *p* < 0.0001) (Figure 6b, Table 3). Post‐hoc Tukey's multiple comparisons (Table 3) revealed that the combination group (HER2‐mRNA‐LNP + RT) exhibited significantly smaller tumor volumes than the LNP control group from Day 3 onward (*p* < 0.05–0.001). Detailed pairwise comparisons and descriptive statistics are provided in Table S3. Tumor volume and weight were significantly smaller in the HER2‐mRNA‐LNP and RT groups than in the LNP group (*p* < 0.01–0.05), and further reduced in the combination group compared with HER2‐mRNA‐LNP alone (*p* < 0.0001–0.001) (Figure 6c,d). In vitro, after 48 h of co‐culture with HER2‐LLC cells, splenic T lymphocytes from the HER2‐mRNA‐LNP and RT groups exhibited significantly higher cytotoxic activity against tumor cells compared with the LNP group (*p* < 0.01–0.05), with the highest killing rate in the combination group (*p* < 0.001) (Figure 6e). Immunohistochemistry demonstrated that both HER2‐mRNA‐LNP and RT significantly reduced HER2 and Ki67 expression in xenograft tumors (*p* < 0.01), and combination therapy resulted in a further decrease (*p* < 0.0001) (Figure 6f,g).

**FIGURE 6:**
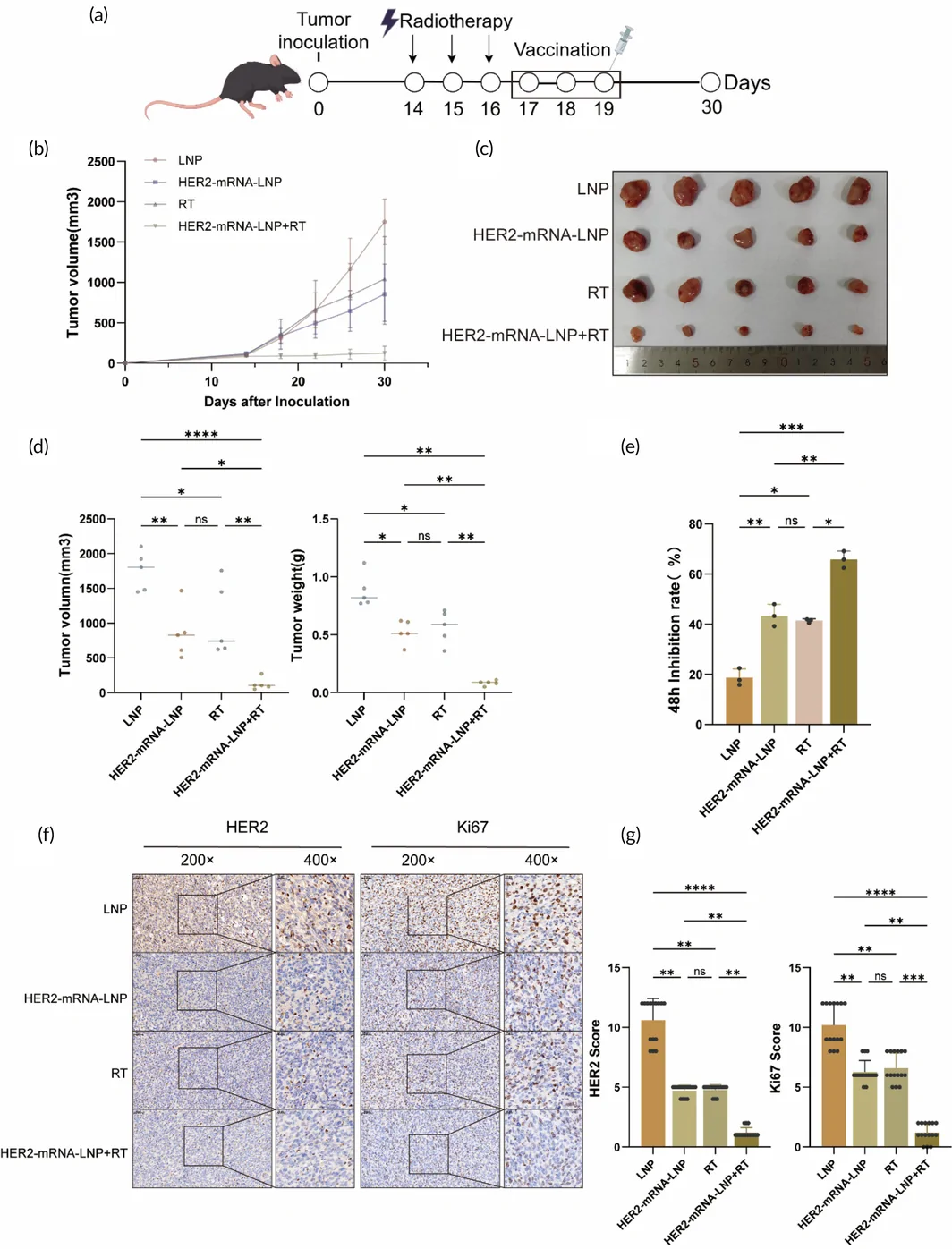
(a) Experimental timeline for each group. (b) Tumor growth curves of HER2‐LLC tumor‐bearing mice. (c) Representative tumor images. (d) Quantification of tumor volume and weight (*n* = 5). (e) CCK‐8 assay‐based cytotoxicity inhibition rate at 48 h (*n* = 3). (f) Representative immunohistochemical images of HER2 and Ki67 expression in mouse tumor tissues. (g) Quantitative analysis of HER2 and Ki67 immunohistochemical staining across groups (Three random fields per mouse were selected for analysis. *n* = 5). All data are presented as mean ± SEM. Two‐way repeated‐measures ANOVA with Tukey's test was used for tumor growth curves (*n* = 5) (b); one‐way ANOVA with Tukey's test was used for multiple group comparisons (d,e,g).

**TABLE 3:** Results of two‐way repeated‐measures ANOVA for tumor volume: therapeutic intervention.

Source of variation	Sum of squares	df	Mean square	*F*‐Value	*p*‐Value	Geisser–Greenhouse *ε*
Group × Time	5,573,960	15, 80	371,597	12.59	<0.0001	–
Time	12,993,686	1.487, 23.80	2,598,737	88.04	<0.0001	0.2975
Group	5,386,521	3, 16	1,795,507	13.66	<0.0001	–
Subject	2,102,871	16, 80	131,429	4.453	<0.0001	–

*Note*: Two‐way repeated measures ANOVA was performed to assess the effects of groups (four groups) (*n* = 5), time, and their interaction on tumor volume, with subjects as the repeated measure. The Geisser–Greenhouse correction was applied for sphericity violation. –, not applicable. ****p < 0.0001.

## 4 DISCUSSION

The remarkable success of mRNA vaccines during the COVID‐19 pandemic has accelerated the maturation of mRNA vaccine technology,^33^, ^34^ promoting its broader application in cancer research and offering a promising avenue for cancer prevention and treatment. mRNA‐LNP vaccines represent a milestone in vaccine technology due to their potential for rapid development, potent immunogenicity, high safety profile, and versatility across multiple fields. mRNA vaccines avoid the risk of genomic integration associated with DNA therapies through transient intracellular protein expression. LNPs, leveraging electrostatic interactions between ionizable lipids and mRNA, enable precise antigen release in vivo based on pH gradients, establishing them as the leading delivery platform in clinical research. As an emerging cancer therapy, only a few LNP‐based mRNA vaccines are currently in clinical studies for lung cancer.^35^, ^36^ In a phase Ib study, Papachristofilou et al.^37^ reported that BI 1361849 (CV9202), an mRNA vaccine encoding five tumor‐associated antigens, was well‐tolerated in combination with RT in stage IV NSCLC patients, with 40% of patients showing at least a two‐fold increase in functional CD4^+^ and CD8^+^ T cells post‐vaccination. In a phase I/IIa study, Sebastian et al.^38^ found that the antigen‐specific immunotherapy CV9201 was well‐tolerated and enhanced immune responses in stage IIIB/IV NSCLC patients. These clinical studies demonstrated the immune activity and safety of mRNA‐LNP vaccines in NSCLC but did not specifically focus on HER2‐overexpressing patients. To our knowledge, this is the first study to apply mRNA‐LNP technology in a mouse model of HER2‐overexpressing NSCLC. We designed an mRNA‐LNP vaccine encoding the HER2 ECD, combining the high efficiency and safety of the LNP platform with the precision of HER2 targeting, thereby addressing a research gap for mRNA‐LNP vaccines in HER2‐overexpressing NSCLC. Furthermore, this study integrates the cutting‐edge approach of combining RT with vaccines in cancer therapy. We first administered the vaccine prophylactically to assess its protective effect and then applied RT to tumor‐bearing mice with pre‐existing HER2‐specific antibodies to study radiosensitization. Additionally, this study is the first to demonstrate that this vaccine combined with RT exerts an enhanced therapeutic effect in NSCLC‐bearing mice and explores potential immune activation mechanisms.

The selection of the HER2‐mRNA‐LNP vaccine dose and radiotherapy parameters in this study was guided by established preclinical and clinical precedents. For the mRNA‐LNP vaccine, a dose of 10 μg per mouse per injection was chosen based on prior reports demonstrating robust immunogenicity and favorable safety profiles with comparable mRNA‐LNP formulations in murine models.^39^, ^40^, ^41^ Considering the LNP composition, mouse strain, intramuscular route of administration, and the therapeutic objective of antitumor vaccination employed in this study, this dose represents an optimal range that balances delivery efficiency, antigen expression levels, immune response intensity, and biosafety. The injection volume of 100 μL is consistent with standard intramuscular administration protocols for lipid nanoparticle‐based vaccines, ensuring adequate dispersion at the injection site while facilitating lymphatic drainage to enhance antigen presentation. The radiotherapy regimen employed in this study, consisting of 8 Gy × 3 fractions (total 24 Gy), was selected based on established preclinical evidence defining the optimal immunomodulatory dose window for hypofractionated radiotherapy in murine tumor models. Both tumor‐control efficacy and the frequency of tumor‐reactive T cells increase with radiation dose per fraction, with maximal immunogenic effects at 7.5–15 Gy per fraction. Notably, 8 Gy in three fractions has been shown to enhance antitumor immune responses when combined with immunotherapy in animal models. Furthermore, this hypofractionated approach aligns with the principles of stereotactic body radiotherapy (SBRT), where moderate hypofractionation (8–12 Gy per fraction) balances effective tumor cytoreduction with preservation of antitumor immunity, minimizing lymphodepletion while maximizing immunogenic cell death. The total dose of 24 Gy delivered in three fractions was selected to achieve robust local tumor control in the HER2‐LLC model while maintaining a favorable immune microenvironment to maximize the antitumor efficacy of the HER2‐mRNA‐LNP vaccine.^42^, ^43^

Of note, all animal experiments in this study were performed using male C57BL/6 mice. Male mice exhibit stable LLC tumor growth and low individual variability, thereby avoiding estrogen‐mediated fluctuations in tumor proliferation and immune status that may confound the evaluation of vaccine‐induced immune responses and tumor radiosensitivity. A single‐sex animal design eliminates hormonal confounders, standardizes experimental baselines, and improves the reproducibility of preclinical results, which is a well‐recognized strategy in tumor immunology and radiotherapy research. Nevertheless, sex‐related immune heterogeneity cannot be fully excluded. Future validation studies in female mice will be conducted to systematically assess the impact of sex on therapeutic outcomes and promote comprehensive clinical translation.

In this study, safety evaluation of the HER2‐mRNA‐LNP vaccine was conducted on day 70 (the experimental endpoint) in tumor‐bearing mouse models, with a primary focus on assessing its long‐term tolerability. Consistent with the timeline of pharmacodynamic evaluation, this endpoint enabled a comprehensive assessment of the vaccine's overall safety profile following the completion of the full immune cycle and the execution of antitumor responses. Pathological analysis demonstrated that mice immunized with HER2‐mRNA‐LNP exhibited intact tissue structures without obvious damage or inflammatory infiltration in the heart, liver, lung, and spleen, and their routine blood parameters, liver and kidney function indicators, as well as cardiac enzymes all remained at normal levels, further validating the favorable safety characteristics of the constructed vaccine and confirming the reliable biosafety of the LNP delivery system reported in previous studies. These findings lay a solid foundation for subsequent mechanistic and therapeutic investigations. Nevertheless, the present study only performed safety assessment at the terminal time point and lacked dynamic histopathological monitoring at the early acute phase and middle immune peak phase post‐immunization, making it impossible to exclude potential transient organ injury or subtle pathological changes induced by early immune activation. Therefore, future work will incorporate multiple detection time points (e.g., 24 h, 7 days, 14 days, and 28 days after the final immunization) to systematically characterize both acute and chronic toxicities, thereby providing more sufficient and comprehensive preclinical evidence to support the clinical translation of the HER2‐mRNA‐LNP vaccine. In addition, we included a PBS group as healthy controls in blood biochemical assays to more accurately evaluate safety. However, the PBS group was not included in the H&E examination, which we acknowledge as a limitation. Nevertheless, combined with previously published literature regarding the safety of LNPs and the present blood biochemistry results, our findings still preliminarily verify the safety of the vaccine. We will add a PBS healthy control group in future investigations for further validation.

We found that the increased spleen weight observed in the LNP control group is likely associated with substantial tumor burden, which enhances splenic immune responses and causes compensatory splenic enlargement. Splenomegaly is a well‐characterized consequence of progressive tumor burden in murine cancer models, driven primarily by systemic expansion and splenic accumulation of myeloid‐derived suppressor cells (MDSCs), which functionally suppress antitumor immunity. In the Lewis lung carcinoma model specifically, tumor‐bearing mice develop marked splenomegaly, with MDSC proportions increasing from baseline 2.6% to 22.1% of splenocytes.^44^ Tumor‐derived factors, including G‐CSF, further exacerbate this pathological myelopoiesis by mobilizing immature myeloid progenitors to the spleen.^45^ In‐depth investigations into these mechanisms will be pursued in future studies.

Following prophylactic vaccination, we detected high levels of HER2‐specific antibodies. Tumor growth rate, volume, and weight were significantly reduced in the HER2‐mRNA‐LNP group. Notably, the LNP control group exhibited weak but detectable cytotoxicity against HER2‐LLC cells in co‐culture assays. Such baseline antitumor activity is attributed to endogenous tumor‐specific T cells in tumor‐bearing mice, which mediate spontaneous and measurable antitumor immunity in murine tumor models without exogenous intervention. Nevertheless, the cytotoxicity of the LNP group was significantly lower than that of all treatment groups, verifying that the robust antitumor immune responses observed in HER2‐mRNA‐LNP monotherapy and combination groups were specifically induced by vaccination and radiotherapy. Splenic lymphocytes from vaccinated mice exhibited increased cytotoxicity against HER2‐LLC cells at 0, 24, 48, and 72 h. Reduced expression of HER2 and Ki67 indicated decreased tumor proliferation and malignancy. These results demonstrate that prophylactic HER2‐mRNA‐LNP vaccination confers significant protection. When RT was applied to vaccinated xenografts, tumor growth inhibition was further enhanced, cytotoxicity increased, and HER2/Ki67 expression decreased further, indicating that HER2‐mRNA‐LNP significantly sensitizes tumors to RT. As shown in Figure 3d, all groups received prophylactic vaccination prior to tumor inoculation, ensuring identical initial antigen stimulation across experimental groups. Radiotherapy was administered after three rounds of antibody detection in the combination group. The slight reduction in antibody levels observed in the combination group may be attributed to individual biological heterogeneity among mice, and no statistically significant difference was detected between groups. This minor fluctuation does not affect our overall conclusions, and further confirms the enhanced antitumor efficacy achieved by the combined regimen of vaccine and radiotherapy. Notably, serum HER2‐specific antibodies increased significantly in both C57BL/6 and BALB/c mice post‐vaccination, suggesting that the designed antigen may cover different MHC backgrounds, indicating potential efficacy across diverse genetic backgrounds—a finding of significant developmental value.

We performed Western blot analysis to confirm human HER2 protein expression in HER2‐LLC cells (Figure 3a). The results demonstrated that HER2‐LLC cells exhibited stable and significantly higher HER2 expression compared with wild‐type LLC cells, with expression levels falling between those of the HER2‐positive breast cancer cell line SKBR3 (positive control) and the moderately HER2‐expressing NSCLC cell line A549. These data validate that the HER2‐overexpressing LLC cell line is a suitable tool for evaluating the in vivo efficacy of HER2‐targeted therapies. Nevertheless, we acknowledge that this artificially modified murine cell line cannot fully recapitulate the biological complexity and heterogeneity of primary human NSCLC tissues. HER2 expression in clinical NSCLC is highly heterogeneous and differs from the constitutive overexpression achieved in this engineered model, and the murine tumor microenvironment lacks human‐specific stromal and immune components, which may also influence HER2 expression patterns and therapeutic responses.

It should be clearly recognized that human HER2 is a xenogeneic antigen in mice, and no endogenous homologous protein is expressed in normal mouse tissues. This characteristic may artificially enhance the overall immunogenicity of the HER2‐targeted vaccine. More importantly, since mice lack physiological human HER2 expression in normal tissues, the current model cannot fully simulate the clinical situation, and thus fails to accurately assess vaccine‐related autoimmune risks and off‐target immune injury to normal tissues. This study primarily adopts this classic murine model to preliminarily explore the immunological mechanism and antitumor activity of the mRNA‐LNP vaccine combined with radiotherapy. It serves as a feasible platform for preclinical efficacy and mechanistic screening. Given the obvious interspecies differences in HER2 expression and immune tolerance, the results obtained from this model cannot be directly extrapolated to clinical application. The translational value of these preclinical findings should be interpreted with caution, and direct clinical inference should be avoided. Further validation using HER2‐transgenic mice, humanized animal models, or clinical samples is required to support clinical translation in future studies. Based on the prophylactic efficacy and radiosensitizing effects of the HER2‐mRNA‐LNP vaccine, we further analyzed changes in the immune microenvironment of tumor tissues and spleens. Further analysis of CD8^+^ T cell subsets revealed that the proportions of CD8^+^ effector memory T cells (Tem) and granzyme B‐positive CD8^+^ T cells were significantly elevated across all treatment groups compared with the control group. These findings indicate that radiotherapy combined with mRNA vaccination effectively activates CD8^+^ cytotoxic T cells and enhances antitumor cellular immunity. The concomitant enrichment of effector memory T cells further suggests that this regimen may induce antigen‐specific immune memory, thereby holding potential for establishing long‐term antitumor immunity. Additional experimental validation is warranted to fully elucidate the sustained immunoprotective effects.

Notably, the proportion of CD44^−^CD62L^−^ double‐negative (DN) T cells was markedly decreased in the radiotherapy group, with a further reduction observed in the combination treatment group. DN T cells represent a terminally differentiated effector subset with a short lifespan that persists poorly in tissues. The concurrent increase in CD8^+^ Tem and granzyme B^+^ cells corroborates this observation, collectively supporting the induction of a substantial bystander activation effect by radiotherapy. Irradiated tumor cells undergo immunogenic cell death, releasing substantial damage‐associated molecular patterns and proinflammatory cytokines. These soluble danger signals extensively activate bystander T cells that were previously in a quiescent state within the tumor microenvironment, thereby reshaping their differentiation trajectory and functional phenotype. On one hand, this process diverts T cells away from differentiation into short‐lived DN effector T cells that exert merely transient cytotoxicity, resulting in a sustained reduction in this subset. On the other hand, it preferentially directs T cell differentiation toward CD8^+^ effector memory T cells with prolonged survival and more stable functionality, accompanied by sustained upregulation of granzyme B expression, thereby conferring robust and durable tumoricidal capacity. The combination of mRNA vaccination with radiotherapy amplifies this radiation‐mediated bystander activation effect, driving T cell differentiation and functional reprogramming to a maximal extent. This is ultimately manifested by the lowest proportion of DN T cells, the highest proportions of Tem and granzyme B‐positive cells, and the most potent T cell‐mediated antitumor immune response in the combination treatment group.

Our results demonstrated that the vaccine effectively activated cellular immunity, as evidenced by a significant increase in HER2‐specific CD8^+^ T cells in both tumor tissues and spleens. Concurrently, elevated B cell proportions were observed in the spleen and tumor microenvironment, coupled with sustained antibody levels, confirming the activation of durable humoral immune responses. However, the absence of significant changes in CD4^+^ T cell infiltration suggests that cell‐mediated cytotoxicity constitutes the predominant effector mechanism, with B cells likely functioning primarily as antigen‐presenting cells to augment CD8^+^ T cell priming rather than directly mediating tumor eradication. Combination with radiotherapy further increased the proportions of CD8^+^ T cells and B cells, indicating modulation of Th1/Th2‐balanced cellular and humoral immunity.

The vaccine group also exhibited elevated NK cell counts and upregulated interferon‐γ (IFN‐γ) expression, which were further amplified by radiotherapy, indicating activation of innate immunity. NK cells can secrete IFN‐γ and directly kill tumor cells with low MHC molecule expression, thereby compensating for blind spots in T cell‐mediated immunity. Additionally, radiotherapy can induce stress ligand expression on tumor cells, enhancing NK cell recognition and cytotoxicity. Increased M1 macrophage proportions and elevated tumor necrosis factor‐α (TNF‐α) levels indicated vaccine‐driven polarization of macrophages toward an antitumor phenotype. Notably, the combination group exhibited a more pronounced increase in splenic M1 macrophages, suggesting that local radiotherapy may induce systemic alterations that promote the transition of tumor‐associated macrophages from the M2 to M1 phenotype.

Concurrent elevation of interleukin‐4 (IL‐4) and IFN‐γ indicated a mixed Th1/Th2 immune response. While increased IL‐4 typically signals Th2‐biased immunity, which may promote regulatory T cell (Treg) expansion and attenuate antitumor effects, Treg cell numbers within the tumor microenvironment were paradoxically decreased in the vaccine‐treated group. This phenomenon may be related to the composition or formulation of our vaccine. In our study, DLin‐MC3‐DMA (MC3) was adopted as the ionizable lipid component, with a molar ratio of 50:10:38.5:1.5 and an N/P ratio of 6:1. A previous study analyzing 325 LNP‐mRNA formulation datasets using the LightGBM algorithm demonstrated that LNPs containing MC3 elicited significantly higher IgG titers in mouse models at an N/P ratio of 6:1, compared with those formulated with alternative ionizable lipids such as SM‐102. The prediction accuracy of the model was high, with the coefficient of determination (*R* ^2^) greater than 0.87. Molecular dynamics simulations further revealed that MC3 lipid molecules efficiently assemble to form a dense nanoparticle core, around which mRNA molecules are wrapped. This structure facilitates optimal encapsulation and pH‐responsive endosomal release of mRNA.^46^ Future studies will focus on optimizing vaccine components, structure, and adjuvant combinations to modulate the Th1/Th2 immune balance and maximize antitumor efficacy.

Collectively, this enhanced antitumor efficacy is predominantly mediated by CD8^+^ T cell‐driven cellular cytotoxicity, with concomitant activation of diverse immune cell populations serving as complementary effector mechanisms.^47^, ^48^, ^49^, ^50^

The observed shift toward M1 macrophage polarization in the HER2‐mRNA‐LNP vaccine group, further amplified in the combination regimen, represents a critical immunological event linking innate inflammatory activation to adaptive tumor clearance. M1 macrophages, classically activated by IFN‐γ and microbial stimuli, exhibit potent antitumor functions through direct cytotoxicity, antigen presentation, and production of proinflammatory cytokines including TNF‐α and IL‐12. Our data demonstrating elevated M1 macrophage proportions and TNF‐α secretion in vaccine‐treated mice indicate that HER2‐mRNA‐LNP vaccination effectively reprograms the tumor‐associated macrophage (TAM) population from the protumoral M2 phenotype toward the antitumoral M1 state. This M1‐dominant polarization serves as a core mechanistic node connecting vaccine‐induced antigen‐specific immunity to tumor suppression: M1 macrophages not only directly phagocytose and kill tumor cells but also efficiently present tumor antigens to CD8^+^ T cells, thereby amplifying the adaptive cytotoxic response. The more pronounced increase in splenic M1 macrophages in the combination group suggests that local radiotherapy may induce a systemic “bystander effect” on myeloid cell compartments, triggering a shift of TAMs from M2 to M1 polarization beyond the irradiation field. This systemic macrophage reprogramming is consistent with emerging evidence that radiotherapy‐induced tumor cell death releases damage‐associated molecular patterns (DAMPs) and tumor antigens, which are subsequently recognized by pattern recognition receptors on macrophages, promoting their M1 functional commitment.

Concurrently, the elevated infiltration and optimized CD8^+^/CD4^+^ T cell ratio in vaccine‐treated groups carry profound implications for adaptive antitumor immunity. The CD8^+^/CD4^+^ ratio serves as a quantitative surrogate for the balance between cytotoxic effector capacity and regulatory/helper functions within the tumor microenvironment. An increased CD8^+^/CD4^+^ ratio indicates enhanced cytotoxic T cell‐mediated tumor killing relative to CD4^+^ T cell‐mediated immunomodulation. In our model, the selective enrichment of CD8^+^ T cells without concomitant CD4^+^ T cell expansion suggests that HER2‐mRNA‐LNP vaccination preferentially elicits antigen‐specific cytotoxic T lymphocyte (CTL) responses rather than CD4^+^ helper‐dependent humoral immunity. This immunophenotypic profile is characteristic of potent cancer vaccines that bypass traditional T cell help requirements through direct cross‐presentation of mRNA‐encoded antigens by professional antigen‐presenting cells, including dendritic cells and M1 macrophages, onto MHC class I molecules. The further optimization of the CD8^+^/CD4^+^ ratio in the combination group reflects radiotherapy‐mediated enhancement of antigen cross‐presentation and MHC class I upregulation on tumor cells, which facilitates more efficient CD8^+^ T cell priming and effector differentiation. Radiotherapy‐induced tumor cell death also expands the available antigen pool and releases type I interferons, creating a positive feedback loop that reinforces CD8^+^ T cell dominance while suppressing regulatory T cell expansion.

Collectively, the coordinated M1 macrophage polarization and CD8^+^/CD4^+^ ratio optimization observed in our study reveal a multimodal immune network orchestrated by the HER2‐mRNA‐LNP vaccine and amplified by radiotherapy. M1 macrophages and CD8^+^ T cells engage in reciprocal positive reinforcement: M1 macrophages produce IL‐12 and present antigens to sustain CD8^+^ T cell proliferation and effector function, while CD8^+^ T cell‐derived IFN‐γ further consolidates M1 polarization and suppresses M2 reprogramming. This self‐amplifying immune axis, established through vaccine priming and radiotherapy boosting, provides a mechanistic foundation for the improved antitumor potency of the combined regimen and offers a rationale for clinical translation in HER2‐overexpressing NSCLC. Furthermore, HER2‐mRNA‐LNP demonstrated therapeutic efficacy in tumor‐bearing mice, significantly reducing tumor growth and enhancing splenic lymphocyte cytotoxicity. RT alone showed comparable effects. Inspired by the PACIFIC trial, this work demonstrates that post‐radiation vaccination elicits enhanced antitumor responses.

However, the clinical translation of mRNA‐LNP vaccines faces several challenges: high tumor antigen diversity and rapid evolution hinder vaccine adaptability; precise targeting is limited by insufficient knowledge of cancer gene loci; although widely used, LNPs require further optimization for biosafety and biocompatibility; and efficacy is constrained by the immunosuppressive tumor microenvironment.^51^

Future studies will focus on the vaccine's long‐term immune protection and memory formation through analysis of memory cell dynamics. To elucidate the underlying mechanisms, experiments such as T cell depletion assays and antibody transfer studies will be conducted, along with efforts to identify the specific epitopes recognized by HER2 antibodies in vivo. Additional work will evaluate its efficacy and safety in other species, optimize dosing and scheduling with radiotherapy, explore combinations with immune checkpoint inhibitors, and elucidate underlying immune mechanisms to support clinical translation. Despite current challenges, the strong prophylactic and therapeutic efficacy, especially when combined with RT, provides a rationale for developing safe and effective strategies for HER2‐overexpressing NSCLC, including relapse prevention and rapid‐response treatment.

## 5 CONCLUSIONS

This study establishes a novel and potent combination strategy integrating a HER2‐targeted mRNA‐LNP vaccine with radiotherapy for HER2‐overexpressing NSCLC. We provide the first evidence that this combination not only suppresses tumor growth in both prophylactic and therapeutic settings but also reshapes the tumor immune landscape by enhancing cytotoxic T cell and NK cell infiltration, promoting M1 macrophage polarization, and inducing a robust systemic Th1/Th2 response. These findings offer a strong preclinical rationale for leveraging mRNA vaccine technology to radiosensitize tumors and overcome immunosuppression.

Despite the promising results, this study has limitations. The use of a syngeneic mouse model may not fully recapitulate the complexity of the human tumor microenvironment and immune system. Future studies employing patient‐derived xenograft (PDX) models or humanized mice would be valuable to strengthen the clinical relevance. Additionally, the long‐term durability of the immune response and the role of immune memory warrant further investigation.

Looking forward, the flexibility of the mRNA‐LNP platform positions it as an ideal candidate for personalized cancer immunotherapy. Future work will focus on identifying optimal neoantigen combinations and refining targeting strategies to enhance tumor specificity and reduce off‐target effects. Ultimately, our findings pave the way for the clinical translation of this radio‐immunotherapy combination, with the goal of improving outcomes for patients with HER2‐positive and other challenging malignancies.

## AUTHOR CONTRIBUTIONS

**Shuo Wang:** Conceptualization; methodology; software; data curation; formal analysis; validation; investigation; writing – original draft; writing – review and editing. **Shaowu Jing:** Writing – review and editing. **Yao Zhang:** Software. **Xinyi Li:** Validation. **Jun Wang:** Project administration; resources; supervision; funding acquisition; writing – review and editing. **Mingming Ge:** Validation. **Lianmei Zhao:** Project administration; resources. **Kangqi Gao:** Validation. **Suyang Zou:** Investigation.

## FUNDING INFORMATION

This work was supported by the Applied Project of Advanced Medical Equipment.

## CONFLICT OF INTEREST STATEMENT

The authors declare no conflicts of interest.

## Supporting information


**Table S1:** Tumor volume (mm^3^) in different treatment groups at various time points (mean ± SEM) and key Tukey's multiple comparison results: preventive treatment.
**Table S2:** Serum HER2 antibody titers (mean ± SD) and Tukey's post‐hoc comparisons between PBS, 1‐dose, and 3‐dose groups.
**Table S3:** Tumor volume (mm^3^) in different treatment groups at various time points (mean ± SEM) and key Tukey's multiple comparison results: therapeutic intervention.
**Figure S1:** Serial flow cytometric gating strategy for multiple immune cell subsets in tumor tissues of tumor‐bearing mice. (a) Stepwise gating to define CD4^+^ helper T cells and CD8^+^ cytotoxic T lymphocytes, CD8^+^ T cells were subsequently gated to identify effector memory T cells, double‐negative T cells and Granzyme B^+^ T cells. (b) Gating for F4/80^+^ macrophages and their CD86^+^ M1 and CD206^+^ M2 polarized subpopulation. (c) Gating of NK1.1^+^ NK cells and CD19^+^ B lymphocytes within the CD45^+^CD3e^−^ non‐T immune compartment. (d) Sequential gating for CD3e^+^CD4^+^CD25^+^FOXP3^+^ regulatory T cells (Tregs). Dead cells were excluded by fixable viability dye staining in all samples. Cell debris and doublets were also excluded during the gating process to ensure data reliability.
**Figure S2:** (a) Representative flow cytometric gating strategy for immune cell subsets derived from mouse spleen. Stepwise identification of CD4^+^ and CD8^+^ T lymphocytes. (b) Gating of F4/80^+^ macrophages and their CD86^−^ labeled M1 and CD206^−^ labeled M2 polarized subpopulations. (c) Gating for NK1.1^+^ NK cells and CD19^+^ B cells from the CD45^+^CD3e^−^ non‐T immune cell population. Dead cells were excluded by fixable viability dye staining in all samples. Cell debris and doublets were also excluded during the gating process to ensure data reliability.

## Data Availability

The data that support the findings of this study are available from the corresponding author upon reasonable request.
