# Supplementary material for: Combined HER2‐targeted mRNA‐LNP vaccine and radiotherapy suppress Lewis lung carcinoma growth in mice: Efficacy and mechanistic insights
Source: Bioeng Transl Med. 2026 Jul 20;11(5):e70160. doi: 10.1002/btm2.70160 (PMC13636190; doi:10.1002/btm2.70160)
Supplement: Supplementary file 1 [file BTM2-11-e70160-s001.docx]

**Supplementary Table 1.**

Tumor volume (mm³) in different treatment groups at various time points (Mean ± SEM) and key Tukey's multiple comparison results: preventive treatment.

| **Time** | **LNP** | **HER2-mRNA-LNP** | **RT** | **HER2-mRNA-LNP+RT** | **Key pairwise comparisons (adjusted P-value)** |
| --- | --- | --- | --- | --- | --- |
| Day 0 | 0.00 ± 0.00 | 0.00 ± 0.00 | 0.00 ± 0.00 | 0.00 ± 0.00 | All pairwise: ns |
| Day 2 | 0.00 ± 0.00 | 0.00 ± 0.00 | 0.00 ± 0.00 | 0.00 ± 0.00 | All pairwise: ns |
| Day 4 | 2.44 ± 1.00 | 0.00 ± 0.00 | 2.44 ± 1.00 | 0.00 ± 0.00 | All pairwise: ns |
| Day 6 | 7.30 ± 1.28 | 0.00 ± 0.00 | 7.32 ± 1.27 | 0.00 ± 0.00 | LNP vs HER2: * (0.0158); HER2 vs RT: * (0.0151); RT vs Combo: * (0.0151) |
| Day 8 | 29.20 ± 4.46 | 1.24 ± 1.24 | 29.20 ± 4.59 | 0.90 ± 0.90 | LNP vs HER2: ** (0.0086); LNP vs Combo: ** (0.0093); HER2 vs RT: ** (0.0098); RT vs Combo: * (0.0104) |
| Day 10 | 62.00 ± 2.55 | 6.70 ± 3.60 | 50.00 ± 11.29 | 5.70 ± 2.61 | LNP vs HER2: **** (<0.0001); LNP vs Combo: **** (<0.0001) |
| Day 12 | 94.40 ± 5.71 | 10.20 ± 4.18 | 63.40 ± 16.39 | 8.86 ± 2.62 | LNP vs HER2: **** (<0.0001); LNP vs Combo: **** (<0.0001) |
| Day 14 | 197.60 ± 42.06 | 21.34 ± 10.05 | 82.93 ± 28.36 | 11.84 ± 3.42 | All pairwise: ns |
| Day 16 | 241.40 ± 22.58 | 39.40 ± 15.81 | 88.30 ± 30.32 | 12.73 ± 3.52 | LNP vs HER2: *** (0.0006); LNP vs Combo: ** (0.0016) |
| Day 18 | 283.20 ± 19.63 | 56.80 ± 19.19 | 93.34 ± 32.88 | 13.64 ± 3.75 | LNP vs HER2: *** (0.0002); LNP vs Combo: *** (0.0004) |
| Day 20 | 400.20 ± 17.92 | 78.00 ± 27.51 | 97.40 ± 32.53 | 14.86 ± 3.94 | LNP vs HER2: *** (0.0001); LNP vs RT: *** (0.0006); LNP vs Combo: **** (<0.0001) |
| Day 22 | 424.40 ± 15.46 | 92.20 ± 31.00 | 113.30 ± 40.50 | 16.08 ± 4.86 | LNP vs HER2: *** (0.0003); LNP vs RT: ** (0.0028); LNP vs Combo: **** (<0.0001) |
| Day 24 | 467.10 ± 29.17 | 102.70 ± 30.87 | 130.80 ± 43.27 | 17.89 ± 5.13 | LNP vs HER2: *** (0.0001); LNP vs RT: ** (0.0015); LNP vs Combo: *** (0.0003) |

SEM, standard error of the mean. Combo = HER2-mRNA-LNP+RT; HER2 = HER2-mRNA-LNP. **P* < 0.05, ***P* < 0.01, ****P* < 0.001, *****P* < 0.0001; ns, not significant. Tukey's test with Geisser-Greenhouse correction. n = 5 per group.

**Supplementary Table 2.**

Serum HER2 antibody titers (Mean ± SD) and Tukey's post-hoc comparisons between PBS, 1-dose, and 3-dose groups.

| **Time** | **PBS** | **1-dose** | **3-dose** | **Key pairwise comparisons (adjusted P-value)** |
| --- | --- | --- | --- | --- |
| Day 0 | 0.00 ± 0.00 | 0.00 ± 0.00 | 0.00 ± 0.00 | All pairwise: ns |
| Day 15 | 0.00 ± 0.00 | 260.00 ± 15.81 | 260.80 ± 17.09 | PBS vs 1-dose: **** (<0.0001); PBS vs 3-dose: **** (<0.0001); 1-dose vs 3-dose: ns (0.9968) |
| Day 30 | 0.00 ± 0.00 | 260.00 ± 15.81 | 695.00 ± 36.40 | PBS vs 1-dose: **** (<0.0001); PBS vs 3-dose: **** (<0.0001); 1-dose vs 3-dose: **** (<0.0001) |
| Day 45 | 0.00 ± 0.00 | 260.00 ± 15.81 | 2250.00 ± 111.80 | PBS vs 1-dose: **** (<0.0001); PBS vs 3-dose: **** (<0.0001); 1-dose vs 3-dose: **** (<0.0001) |
| Day 70 | 0.00 ± 0.00 | 195.00 ± 11.18 | 2233.80 ± 126.74 | PBS vs 1-dose: **** (<0.0001); PBS vs 3-dose: **** (<0.0001); 1-dose vs 3-dose: **** (<0.0001) |

Data are presented as mean ± standard deviation (SD). Key pairwise comparisons show adjusted P-values from Tukey's test with Geisser-Greenhouse correction. *****P* < 0.0001; ns, not significant (*P* > 0.05). n = 5 per group. Antibody titers are expressed as ng/mL.

**Supplementary Table 3.**

Tumor volume (mm³) in different treatment groups at various time points (Mean ± SEM) and key Tukey's multiple comparison results: therapeutic intervention

| **Time** | **LNP** | **HER2-mRNA-LNP** | **RT** | **HER2-mRNA-LNP+RT** | **Key pairwise comparisons (adjusted P)** |
| --- | --- | --- | --- | --- | --- |
| Day 0 | 0.00 ± 0.00 | 0.00 ± 0.00 | 0.00 ± 0.00 | 0.00 ± 0.00 | All pairwise: ns |
| Day 14 | 99.43 ± 4.72 | 116.60 ± 9.08 | 108.60 ± 14.57 | 83.44 ± 4.97 | All pairwise: ns |
| Day 18 | 308.40 ± 25.86 | 336.30 ± 43.12 | 359.00 ± 83.05 | 89.27 ± 14.34 | LNP vs Combo: ** (0.0011); HER2 vs Combo: * (0.0114) |
| Day 22 | 655.50 ± 96.89 | 495.80 ± 60.37 | 666.90 ± 160.10 | 92.23 ± 15.05 | LNP vs Combo: ** (0.0011); HER2 vs Combo: * (0.0115) |
| Day 26 | 1168.00 ± 169.60 | 647.10 ± 112.70 | 837.10 ± 178.00 | 110.90 ± 27.40 | LNP vs Combo: ** (0.0011); HER2 vs Combo: * (0.0116) |
| Day 30 | 1752.00 ± 126.40 | 855.30 ± 166.90 | 1042.00 ± 234.80 | 124.90 ± 38.41 | LNP vs Combo: ** (0.0011); HER2 vs Combo: * (0.0117) |

SEM, standard error of the mean. Combo = HER2-mRNA-LNP^+^RT; HER2 = HER2-mRNA-LNP. **P* < 0.05, ***P* < 0.01, ****P* < 0.001; ns, not significant. Tukey's test with Geisser-Greenhouse correction. n = 5 per group.

**Supplementary Figure 1.**

Serial flow cytometric gating strategy for multiple immune cell subsets in tumor tissues of tumor-bearing mice. Stepwise gating to define CD4^+^ helper T cells and CD8^+^ cytotoxic T lymphocytes, CD8^+^ T cells were subsequently gated to identify effector memory T cells, double-negative T cells and Granzyme B^+^ T cells (A). Gating for F4/80^+^ macrophages and their CD86^+^ M1 and CD206^+^ M2 polarized subpopulations (B). Gating of NK1.1^+^ NK cells and CD19^+^ B lymphocytes within the CD45^+^CD3e^-^ non-T immune compartment (C). Sequential gating for CD3e^+^CD4^+^CD25^+^FOXP3^+^ regulatory T cells (Tregs) (D). Dead cells were excluded by fixable viability dye staining in all samples. Cell debris and doublets were also excluded during the gating process to ensure data reliability.

**Supplementary Figure 2.**

Representative flow cytometric gating strategy for immune cell subsets derived from mouse spleen. Stepwise identification of CD4^+^ and CD8^+^ T lymphocytes (A). Gating of F4/80^+^ macrophages and their CD86^-^labelled M1 and CD206^-^labelled M2 polarized subpopulations (B). Gating for NK1.1^+^ NK cells and CD19^+^ B cells from the CD45^+^CD3e^-^ non-T immune cell population (C). Dead cells were excluded by fixable viability dye staining in all samples. Cell debris and doublets were also excluded during the gating process to ensure data reliability.

**Supplementary Figure 1**

**Supplementary Figure 2**
